# ILF3 Regulates Cell Proliferation and Metastasis by Competitively Antagonizing the Interaction Between HMGCL and USP38 in Hepatocellular Carcinoma

**DOI:** 10.1155/humu/2654435

**Published:** 2026-04-29

**Authors:** Qingqing Luo, Lei Xiao, Ganlu Deng, Tan Deng, Wenchao Zhao, Rensheng Wang, Xueying Hu

**Affiliations:** ^1^ Department of Radiation Oncology & Guangxi Key Laboratory of Enhanced Recovery After Surgery for Gastrointestinal Cancer, The First Affiliated Hospital of Guangxi Medical University, Nanning, Guangxi, China, gxmu.edu.cn; ^2^ Department of Oncology, Hunan Provincial People’s Hospital, The First Affiliated Hospital of Hunan Normal University, Changsha, Hunan Province, China, hunnu.edu.cn; ^3^ Department of Colorectal Surgery, Hunan Cancer Hospital/Affiliated Cancer Hospital of Xiangya School of Medicine, Central South University, Changsha, Hunan Province, China, csu.edu.cn; ^4^ Department of Oncology, The First Affiliated Hospital of Guangxi Medical University, Nanning, Guangxi, China, gxmu.edu.cn

**Keywords:** deubiquitinase, HCC, HMGCL, ILF3, USP38

## Abstract

**Background:**

Hepatocellular carcinoma (HCC) is a major type of primary liver cancer. Previous studies have reported that interleukin enhancer–binding factor 3 (ILF3) is involved in the regulation of multiple cancers. This study investigated the molecular mechanisms whereby ILF3 promotes HCC progression.

**Methods:**

ILF3 expression levels were determined through immunohistochemistry (IHC) and Western blot (WB) analyses. The biological functions of ILF3 in HCC were evaluated using both in vitro assays and in vivo animal models. Co‐immunoprecipitation (Co‐IP) was carried out to identify HMGCL as a binding partner of ILF3. To clarify the potential molecular pathways underlying ILF3‐mediated regulation of HCC malignant behaviors, protein stability assays and in vitro ubiquitination experiments were performed.

**Results:**

ILF3 was significantly upregulated in HCC. The patients with high expression of ILF3 showed poor prognosis in our cohort. ILF3 knockdown inhibited the proliferation and metastasis of HCC cells both in vitro and in vivo in this study. Mechanistically, ILF3 was found to be bound to HMGCL and to accelerate its protein degradation. Additionally, we found that ILF3 promotes HCC cell proliferation and metastasis through HMGCL. Overexpression of HMGCL in ILF3‐upregulated HCC cells could significantly reverse the proliferation and invasion role of ILF3 on HCC cells. Moreover, USP38 was identified as a deubiquitinating enzyme that participates in promoting the stability of HMGCL. ILF3 disrupted the interaction between USP38 and HMGCL, thereby enhancing HMGCL ubiquitination and accelerating its degradation.

**Conclusion:**

ILF3 promotes the proliferation and metastasis of HCC by enhancing the ubiquitination of HMGCL by interfering with the interaction between the deubiquitinase USP38 and HMGCL.

## 1. Introduction

Hepatocellular carcinoma (HCC) accounts for a predominant subtype of primary liver cancer, frequently linked to chronic liver disease and cirrhosis [1, 2]. HCC also serves as one of the top contributors to cancer‐related mortality in the world. There are approximately 830,000 new patients diagnosed with HCC each year [3–6]. In current clinical practice, molecularly targeted therapy has brought about substantial improvements in treatment efficacy for HCC patients [7, 8]. Nevertheless, the molecular mechanisms underlying HCC tumorigenesis and progression remain incompletely understood. Given its high proliferative rate and strong metastatic potential, many HCC patients are diagnosed when the disease has already advanced to later stages [9, 10]. There is a crucial task for us to identify effective molecular biomarkers to facilitate early detection of HCC. We need to develop new therapeutic strategies to enhance the prognosis of patients with HCC.

ILF3 (interleukin enhancer–binding factor 3) is famous as a transcription factor for some protein molecules. There were some research studies reporting that ILF3 functions as a negative modulator of innate immune responses [11–13]. ILF3 was a double‐stranded RNA‐binding protein involved in a wide range of cellular processes and pathways [14,15]. ILF3 is a ubiquitous and abundant protein that can shuttle between the nucleus and cytoplasm of the cell. The phosphorylation status of ILF3 drives its expression in cancer cells [16]. Accumulating evidence has demonstrated that ILF3 is an oncogenic driver in regulating tumor proliferation. For instance, ILF3 is frequently upregulated in colorectal cancer, serving as a substrate for SPOP and modulating serine biosynthesis [17]. Additionally, ILF3 has been shown to regulate vascular endothelial growth in breast cancer and to govern the cell cycle in HCC cells [18, 19]. Despite growing evidence supporting ILF3’s oncogenic role across various tumors, the exact molecular pathways through which ILF3 modulates HCC proliferation and metastasis remain incompletely elucidated.

In the current study, we revealed that ILF3 is significantly upregulated in HCC. We also manipulated ILF3 expression in HCC and explored the function of ILF3 in proliferation and metastasis through in vitro and in vivo assays. Mechanistically, we provided evidence to uncover the potential molecular mechanism of ILF3 in HCC, by which ILF3 interacts with HMGCL and downregulates its expression by promoting its ubiquitination. Additionally, we demonstrated that USP38 also interacts with HMGCL and contributes to HMGCL deubiquitination. ILF3 competitively antagonizes the interaction between USP38 and HMGCL, thereby reducing USP38‐mediated HMGCL deubiquitination and decreasing HMGCL protein abundance. Our results reveal a novel function of ILF3 in regulating cell proliferation and metastasis and provide new insights into the prevention of HCC metastasis.

## 2. Materials and Methods

### 2.1. Bioinformatic Analysis

The expression data for ILF3 in cancer were downloaded from TCGA and GEPIA [20, 21]. We also performed Gene Set Enrichment Analysis (GSEA) based on the expression levels of ILF3 in HCC from the TCGA gene profile [22]. The expression of ILF3 in HCC was treated as a binary variable and categorized as low or high ILF3 expression based on the median value of ILF3 expression. The difference between the mean values of samples with low and high ILF3 expression was used for GSEA.

### 2.2. Clinical Samples

Clinical HCC tissue samples were obtained from the Pathology Department of the Hunan Provincial People’s Hospital, The First Affiliated Hospital of Hunan Normal University. All study procedures were approved by the Ethics Committee of the Hunan Provincial People’s Hospital. Informed consent was obtained from all patients involved in this study.

### 2.3. Immunohistochemistry (IHC) Assays

Tissue sections were prepared from HCC samples. The sections were heated at 60°C for 2 h, dewaxed with xylene, and hydrated using a graded alcohol series (100%, 95%, 90%, and 75%), followed by three washes with PBS for 5–10 min each. Antigen retrieval was performed by heating the sections in citric acid solution for 8 min. Sections were blocked with 5% goat serum for 1–2 h, washed, and incubated with an anti‐ILF3 antibody at 4°C overnight. Afterward, the sections were washed three times with PBS for 5–10 min each and incubated with a secondary anti‐rabbit antibody at 37°C for 1 h. The sections were stained with a DAB mixture for 5–10 s, counterstained, dehydrated, and cleared. The samples were observed under a microscope, and the expression of ILF3 and HMGCL appeared as yellowish‐brown staining.

### 2.4. Cell Culture and Transfection

The human HCC cell lines Hep3B, HepG2, and Huh7 were used in this study. All cell lines were derived from *Homo sapiens* (human). The sex, tissue of origin, official cell line names, and Research Resource Identifiers (RRIDs) are summarized as follows: (1) Hep3B: male, HCC, RRID: CVCL_0326; (2) HepG2: male, HCC, RRID: CVCL_0027; and (3) Huh7 (official name: Huh‐7): *H. sapiens* (human), male, HCC, RRID: CVCL_0336. The cell lines used in this study were purchased from the Cell Bank of the Chinese Academy of Sciences (Shanghai, China). Prior to experimental use, the identity of each cell line was authenticated by short tandem repeat (STR) profiling. A ≥ 98% match result between the tested profile and the reference profile of each cell line confirmed the authenticity of the cell lines. None of the cell lines used in this study are listed as misidentified or contaminated in the International Cell Line Authentication Committee (ICLAC) Misidentified Cell Lines Database or other relevant public resources. Mycoplasma contamination was tested using a PCR‐based mycoplasma detection kit prior to and during the experimental period. The results confirmed that all cell lines were free of mycoplasma contamination throughout the described experiments.

Cells were cultured in DMEM (Invitrogen, United States) supplemented with 10% fetal bovine serum (Invitrogen, United States) and incubated at 37°C in a 5% CO_2_ incubator. Cells were stored at −80°C as a backup. The ILF3 knockdown and overexpression recombinant lentiviruses were purchased from Genechem (Shanghai, China) to construct stable transfection cell lines. Cells were infected with lentiviral reagents for 6–8 h and then selected with 2 *μ*g/mL puromycin for 2 weeks. The transfection efficiency was evaluated using Western blotting (WB). Full‐length HMGCL and ILF3 siRNA were designed by Shanghai Sangon. Lipofectamine 2000 (Invitrogen, United States) was used for cell transfection.

### 2.5. WB Assay and Co‐Immunoprecipitation (Co‐IP)

Proteins isolated from tissues or cells were separated via SDS–PAGE and transferred onto a polyvinylidene difluoride (PVDF) membrane. The membrane was blocked with 5% skimmed milk for 1 h and incubated with a primary antibody solution at 4°C overnight. The membrane was washed with TBST buffer and incubated with a secondary antibody for 1 h. Protein bands were visualized using enhanced chemiluminescence (ECL) (Advansta, United States). ImageJ was used to measure the grayscale values of WB bands and to quantify the WB results. As for Co‐IP, the cell lysates and a target‐specific antibody were incubated with Protein A/G Plus‐Agarose at 4°C overnight. They were then washed thrice with a specific buffer. Next, the bead–antibody complexes were suspended in the protein lysate and analyzed using WB. The antibodies used in this study are listed in Supporting Information 2: Table S1.

### 2.6. Cell Counting Kit‐8 (CCK‐8) Assays

Cell proliferation was measured using the CCK‐8 assay kit (Yeasen, Shanghai, China). Cells were resuspended and cultured in 96‐well plates. Cells were incubated with 10 *μ*L of CCK‐8 reagent for 2 h, following the manufacturer’s protocol. The absorbance was measured at 450 nm using an Infinite 200 PRO microplate reader (Tecan, Switzerland). The wavelength was set at 450 nm, and the bandwidth was set at 9 nm. The settle time was set at 0 ms to evaluate cell proliferation.

### 2.7. Colony Formation Assay

Approximately 500 cells were plated in 6‐well culture plates and incubated for 2 weeks. The cells were fixed with 4% paraformaldehyde for 20 min and stained with 1% crystal violet for 10–15 min. Colonies were observed under a microscope, and the number of colonies was quantified.

### 2.8. Wound Healing Assay

Cells were plated at the same density in 6‐well plates and cultured until they reached 90% confluence. The cell layers were scratched using a sterile 100‐*μ*L pipette tip to create wound gaps. The cells were then washed with PBS and cultured for 36 h. Wound gaps were photographed at specific time points.

### 2.9. Transwell Assay

Matrigel was applied to the bottom of the Transwell chamber. Approximately 2 × 10^6^ cells/mL were plated into the Transwell chamber and cultured in DMEM. Additionally, 10% fetal bovine serum was added to the 24‐well culture plates. Cells were cultured for 36–48 h. Next, the cells on the upper membrane were removed, and the remaining cells were fixed with 4% paraformaldehyde for 20 min. The cells were stained with 1% crystal violet for 10–15 min and observed under a microscope.

### 2.10. RNA Isolation and RT‐qPCR

RNA was isolated using TRIzol reagent (Invitrogen, Carlsbad, California, United States). RNA was eluted and reverse transcribed using a Reverse Transcription Reagent Kit (Yeasen, Shanghai, China). Real‐time PCR analysis was performed using the SYBR Green Master Mix (Yeasen, Shanghai, China). The results were normalized to GAPDH expression levels. The primers used in this study are listed in Supporting Information 3: Table S2.

### 2.11. Nude Mouse Xenograft Experiments

Animal experiments were reviewed and approved by the Medical Experimental Animal Care Commission of Guangxi Medical University. Adult male nude mice were maintained in a specific pathogen‐free (SPF) facility and were purchased from Vital River (Beijing, China). Cells infected with ILF3‐overexpressing lentivirus were subcutaneously injected below the foreleg on each side. Tumor growth was monitored daily. Tumor volume (*V*) was calculated using the following formula: *V* (mm^3^) = 0.5 × width^2^ × length. In the lung metastasis experiment, mice were injected with 2 × 10^6^ cells per mouse via the tail vein. The mice were sacrificed at a predefined endpoint, and their lungs were removed and evaluated using a double‐blind method. The tumors and whole lungs were fixed in 4% paraformaldehyde and embedded in paraffin. The metastatic nodules in the lungs were counted under a microscope.

### 2.12. Statistical Analysis

Statistical analyses were performed using GraphPad Prism 8.0 and SPSS 25.1 software. Differences between the two groups were analyzed using Student’s *t*‐test. The impact of ILF3 expression on survival was assessed using Kaplan–Meier plots and log‐rank tests. Chi‐square tests were used to examine the associations between ILF3 expression and clinicopathological characteristics.

## 3. Results

### 3.1. ILF3 Is Upregulated in HCC and Associated With Progression of HCC

**Figure 1:**
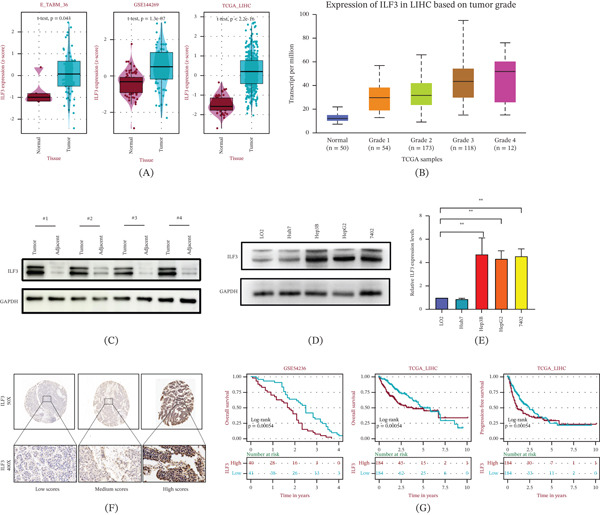
ILF3 expression is significantly upregulated and associated with poor prognosis in hepatocellular carcinoma. (A) ILF3 expression was analyzed utilizing GSE14429 and TCGA. (B) The relationship between the expression of ILF3 in HCC and tumor grade was analyzed using the UALCAN database based on TCGA. (C) Western blot assay was performed to detect the protein levels of ILF3 in HCC and adjacent normal tissues. (D) The protein expression levels of ILF3 detected via Western blot in HCC cell lines and immortalized human normal liver cells. (E) Relative ILF3 levels in HCC cell lines and immortalized human normal liver cells. (F) Immunohistochemistry was performed to detect ILF3 expression in HCC and adjacent normal tissues. (G) The association between ILF3 expression and overall survival and progression‐free survival was analyzed utilizing TCGA and GSE54236. ∗*p* < 0.05, ∗∗*p* < 0.01.

To investigate the potential role of ILF3 in cancer, we first analyzed the expression levels of ILF3 in pan‐cancer. Based on TCGA, it was shown that ILF3 was upregulated at both the RNA and protein levels in most tumors (Supporting Information 1: Figure S1A,B). These findings indicated that ILF3 may participate in the regulation of multiple malignant tumors. Another analysis from GSE14429 and TCGA revealed that ILF3 was significantly upregulated in HCC tissues (Figure 1A). There were consistent observations noted in GEPIA and TCGA (Supporting Information 1: Figure S1C,D). Notably, TCGA database analysis displayed that the expression of ILF3 in HCC showed a significant association with tumor grade (Figure 1B). We subsequently performed WB assays to test ILF3 expression in HCC patient tissues, and we found that the expression of ILF3 levels in HCC tumor tissues was upregulated compared with matched adjacent nontumor tissues (Figure 1C). Similarly, elevated ILF3 levels were demonstrated in most HCC cell lines at both the protein and RNA levels (Figure 1D,E). These data support that ILF3 is closely linked to HCC development.

Further IHC results revealed high ILF3 expression in the majority of samples (Figure 1F). We also investigated the relationship between ILF3 expression in HCC tissues and HCC patients’ clinicopathological characteristics. Notably, high ILF3 expression was significantly associated with tumor size (*χ* ^2^ = 12.242, *p* < 0.01) and TNM stage (*χ* ^2^ = 7.650, *p* < 0.01) (Table 1). Data from TCGA and GSE54236 revealed that patients with high ILF3 levels had poor overall survival and progression‐free survival (Figure 1G). Overall, these results suggested that ILF3 is highly expressed in patients with HCC and is associated with poor prognosis.

**Table 1:** The relationship between the expression of ILF3 and clinicopathological variables of HCC patients (*N* = 88).

ILF3 expression					
Characteristic	Total	Low *n* = 31	High *n* = 57	*χ* ^2^	*p*
Gender					
Male	74	27	47	0.323	0.570
Female	14	4	10		
Tumor size					
≥ 5	58	13	45	12.242	< 0.01∗∗
< 5	30	18	12		
HBs Ag					
Yes	68	21	46	1.083	0.298
No	20	9	11		
Tumor number					
Single	70	26	44	0.550	0.458
Multiple	18	5	13		
Portal vein tumor thrombus (PVTT)					
Yes	20	8	12	0.258	0.611
No	68	23	45		
pTNM stage					
I&II	57	26	31	7.650	< 0.01∗∗
III&IV	31	5	26		
Lymph node metastasis					
Yes	9	4	5	0.373	0.541
No	79	27	52		
Relapse					
Yes	33	11	22	0.083	0.773
No	55	20	35		
AFP					
≤ 400	50	18	32	0.030	0.862
> 400	38	13	25		

∗*p* < 0.05, ∗∗*p* < 0.01.

### 3.2. ILF3 Promotes Proliferation, Invasion, and EMT of HCC In Vitro

**Figure 2:**
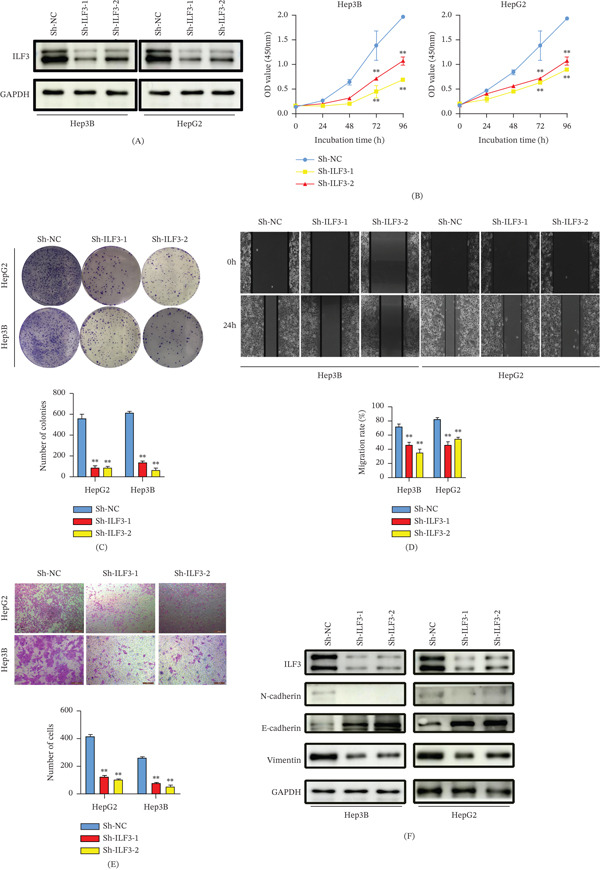
ILF3 promotes cell proliferation and EMT of hepatocellular carcinoma in vitro. (A) The expression levels of ILF3 were detected using Western blotting after transfection with lentivirus‐mediated shRNA. (B) CCK‐8 assays to assess cell proliferation after ILF3 knockdown in Hep3B and HepG2. (C) The colony formation assays were used to measure the colony‐forming ability of HCC cells after transfection with ILF3 knockdown shRNA. (D) The migration of Hep3B and HepG2 measured via wound healing assays after ILF3 knockdown. (E) The invasion of Hep3B and HepG2 measured via Transwell assays after ILF3 knockdown. (F) Western blot assays to evaluate E‐cadherin, N‐cadherin, and vimentin expression in Hep3B and HepG2 cells after ILF3 knockdown. ∗*p* < 0.05, ∗∗*p* < 0.01.

To explore the oncogenic role of ILF3 in HCC, we established stable ILF3 knockdown in Hep3B and HepG2 cells. The transfection efficiency was confirmed via WB analysis (Figure 2A). The CCK‐8 assay showed that silencing ILF3 significantly inhibited cell proliferation (Figure 2). B Colony formation assays revealed that ILF3 knockdown inhibited colony formation in both Hep3B and HepG2 cells (Figure 2C). Additionally, wound healing and Transwell assays were performed to investigate the effect of ILF3 on HCC cell invasion, and it was found that ILF3 knockdown impaired cell migration and invasion (Figure 2D,E). As EMT‐related proteins participate in tumor cell migration and invasion, we examined the protein levels of N‐cadherin, vimentin, and E‐cadherin in cells with ILF3 knockdown. The results showed that ILF3 knockdown significantly increased E‐cadherin expression and reduced N‐cadherin and vimentin expression (Figure 2F).

### 3.3. ILF3 Regulates In Vivo Growth and Metastasis of HCC

**Figure 3:**
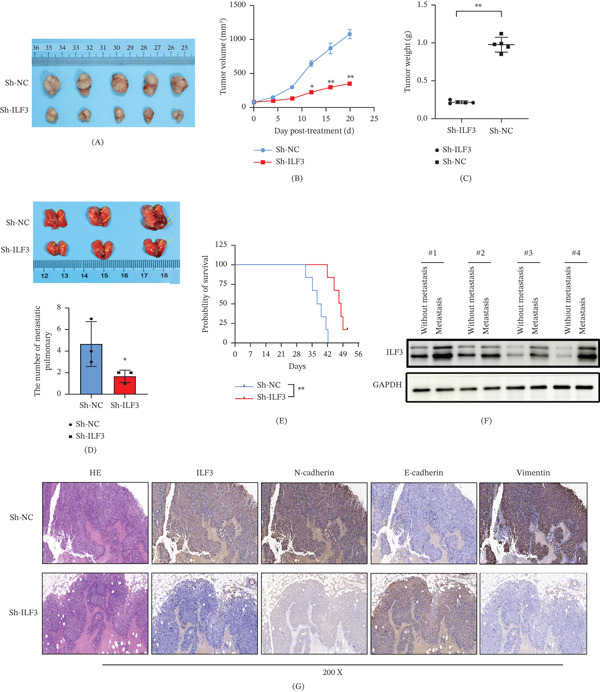
ILF3 regulates in vivo growth and metastasis of hepatocellular carcinoma. (A) Tumors derived from xenograft mice were photographed. (B, C) The volume and weight of the tumor were measured after transfection with ILF3 knockdown shRNA. (D) The lung metastatic tumors derived from xenograft mice were photographed, and the number of lung metastatic nodules was recorded. (E) The survival time was monitored after injecting the ILF3‐knockdown HepG2 cells. (F) Western blot assay was performed to detect the expression of ILF3 in lung metastatic tissues derived from xenograft mice. (G) The expression of ILF3, E‐cadherin, N‐cadherin, and vimentin was measured in lung metastatic tissues using an immunohistochemistry assay. ∗*p* < 0.05, ∗∗*p* < 0.01.

In vivo tumorigenesis experiments were performed. We injected stable ILF3‐knockdown HepG2 cells (Sh‐ILF3) and their control cells (Sh‐NC) into nude mice to explore the effects of ILF3 on HCC tumorigenesis in vivo. The tumor volume and size in nude mice were monitored. Our data indicated that the tumor volume and size in nude mice significantly declined after ILF3 knockdown (Figure 3A,B). Most notably, tumors derived from the ILF3 knockdown group seemed smaller than tumors derived from the negative control group (Figure 3). C We next injected cells from both groups into the tail veins of nude mice. We observed that the number of pulmonary metastatic lesions in the ILF3 knockdown group was less than that in the negative control group. This indicated that ILF3 would promote HCC tumor metastasis (Figure 3D). Furthermore, we monitored the survival time of nude mice in both groups. Mice in the ILF3 knockdown group exhibited a longer survival time (Figure 3E). Additionally, WB analysis revealed that metastatic tissues had much higher ILF3 expression in the Sh‐NC group (Figure 3F). These results demonstrated that ILF3 promotes HCC proliferation and metastasis in vivo. We also performed an IHC assay to detect the expression of N‐cadherin, E‐cadherin, and vimentin in tumors derived from nude mice. Higher N‐cadherin and vimentin expression was observed in the Sh‐ILF3 group (Figure 3G). This indicated that the knockdown of ILF3 expression significantly impaired the metastatic ability of HCC in vivo.

### 3.4. ILF3 Regulates HMGCL Degradation in a Ubiquitin‐Proteasome–Dependent Manner

**Figure 4:**
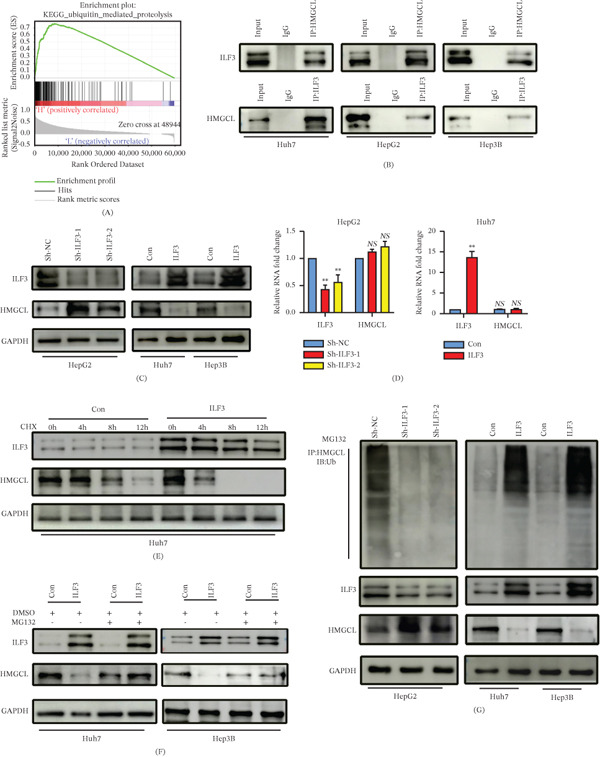
ILF3 interacts with HMGCL and promotes its degradation in a ubiquitin‐proteasome–dependent manner. (A) GSEA comparing the ILF3 low (blue) and high (red) expression subgroups of patients with HCC in TCGA indicated that the high expression of ILF3 showed a significant association with ubiquitin‐mediated proteolysis. (B) Co‐immunoprecipitation (Co‐IP) assay performed to show the interaction between ILF3 and HMGCL in Huh7, HepG2, and Hep3B cells. (C) Western blot assays showed that ILF3 decreased the expression of HMGCL in HCC cells. (D) RT‐qPCR showed that ILF3 had no effect on HMGCL mRNA levels. (E) The expression of HMGCL was measured in ILF3‐overexpressing cells and control cells after treatment with 50 *μ*g/mL cycloheximide. (F) The expression of HMGCL was measured in ILF3‐overexpressing cells and control cells via Western blot in Huh7 and Hep3B cells after treatment with 5 *μ*M MG132. (G) Co‐IP and Western blot were performed to measure the endogenous ILF3 ubiquitination in cells treated with 5 *μ*M MG132. ∗*p* < 0.05, ∗∗*p* < 0.01. Abbreviation: NS, not significant.

To further investigate the mechanisms by which ILF3 promotes proliferation and metastasis in HCC, we first performed GSEA based on TCGA and stratified the samples by the median ILF3 expression in HCC in TCGA. GSEA revealed that “ubiquitin‐mediated proteolysis” was one of the most significantly correlated pathways in the high ILF3 expression group compared with the low ILF3 expression group (Figure 4A). Therefore, we hypothesized that ILF3 regulates HCC proliferation and metastasis by modulating the ubiquitination of specific proteins. Next, we performed Co‐IP assays in HCC cells and identified proteins that interacted with ILF3 using mass spectrometry (Table 2). HMGCL was one of the most abundant proteins in the ILF3 Co‐IP extracts; therefore, it was selected for further investigations. Co‐IP confirmed that HMGCL physically interacted with ILF3 in HCC cells (Figure 4B). Moreover, we found that HMGCL expression was significantly decreased in ILF3 knockdown cells, whereas it was upregulated in ILF3‐overexpressing cells (Figure 4C). However, RT‐qPCR assays showed that ILF3 did not alter HMGCL mRNA levels (Figure 4D), indicating that ILF3 regulates HMGCL protein levels without affecting HMGCL mRNA expression. We extracted cellular proteins from ILF3‐overexpressing Huh7 cells and treated them with cycloheximide (CHX) at different time points. Protein degradation assays indicated that ILF3 overexpression significantly decreased the half‐life of HMGCL (Figure 4E). Additionally, WB analysis revealed that MG132, a proteasome inhibitor, abolished the downregulation of HMGCL expression by ILF3 in Huh7 and Hep3B cells (Figure 4F). To explore whether ILF3 downregulates HMGCL in a ubiquitin‐dependent manner, we performed an in vitro ubiquitination assay. The results showed that the ubiquitination level of HMGCL was significantly increased in ILF3‐overexpressing Huh7 and Hep3B cells. Conversely, the knockdown of ILF3 significantly decreased the ubiquitination of HMGCL in HepG2 cells (Figure 4G). These results suggested that ILF3 enhances HMGCL degradation via a ubiquitin‐proteasome–dependent pathway.

**Table 2:** Mass spectrometry of the top 10 proteins extracted by Co‐IP from ILF3.

No.	Protein names	Gene names	LFQ intensity IgG (nuclear protein)	LFQ intensity ILF3 (nuclear protein)
1	Polyadenylate‐binding protein 2	PABPN1	0	61565000
2	HMG‐CoA‐2 lyase	HMGCL	0	33842000
3	GTP‐binding nuclear protein Ran	RAN	0	26828000
4	ATP‐dependent RNA helicase A	DHX9	0	17028000
5	MBL‐associated serine protease 2	MASP2	0	12166000
6	Protein MAL2	MAL2	0	11381000
7	Retinol‐binding protein 4	RPB4	0	8461700
8	Interleukin enhancer‐binding factor 2	ILF2	0	8107800
9	AMP deaminase 2	AMPD2	0	8026300
10	Elongation factor 1‐beta	EEF1B2	0	7901300

### 3.5. HMGCL Overexpression Alleviates the Role of ILF3 in HCC

**Figure 5:**
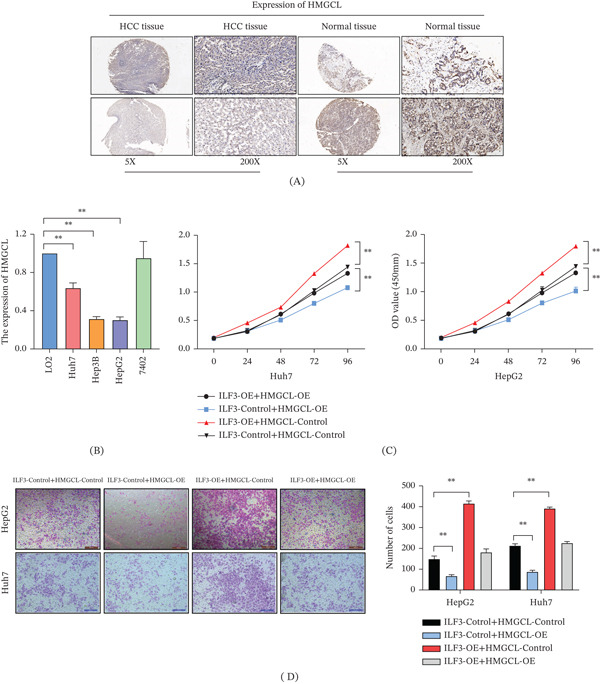
ILF3 promotes proliferation and invasion of hepatocellular carcinoma by inhibiting HMGCL expression. (A) Immunohistochemistry was performed to detect HMGCL expression in HCC and adjacent normal tissues. (B) Relative HMGCL levels in HCC cell lines and immortalized human normal liver cells. (C) CCK‐8 assay was performed to measure cell proliferation in Huh7 and HepG2 cells overexpressing ILF3 and control cells transfected with HMGCL‐overexpressing plasmids. (D) Transwell assay was performed to measure cell proliferation in Huh7 and HepG2 cells overexpressing ILF3 and control cells transfected with HMGCL‐overexpressing plasmids. ∗*p* < 0.05, ∗∗*p* < 0.01.

Further IHC analysis suggested that HMGCL was significantly downregulated in HCC tissues (Figure 5A). This indicates that the dysregulation of HMGCL expression in HCC may be a key factor in the occurrence of HCC. We also performed RT‐qPCR assays and found that HMGCL expression was decreased in HCC cells (Figure 5B). We speculated that ILF3 regulates cell proliferation and metastasis in HCC by inhibiting HMGCL expression. To verify this hypothesis, we transfected the HMGCL‐overexpressing plasmids and their control plasmids into HCC cells with stable overexpression of ILF3 in Huh7 and HepG2 cells. CCK‐8 assays suggested that ILF3 significantly promoted the proliferation of HCC cells, as indicated in previous results. However, cell proliferation was impaired by transfection with HMGCL‐overexpressing plasmids (Figure 5C). Similar results were observed in the Transwell assay (Figure 5D). These results indicate that ILF3 regulates cell proliferation and metastasis by downregulating HMGCL expression.

### 3.6. ILF3 Promotes HMGCL Ubiquitination by Competitively Binding to Deubiquitinase USP38

**Figure 6:**
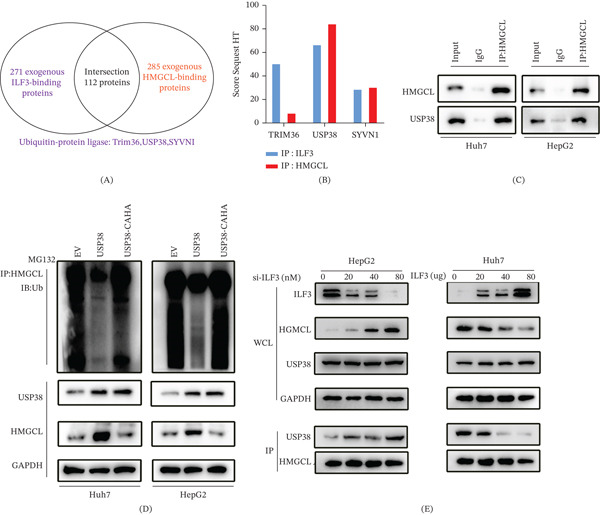
ILF3 promotes HMGCL ubiquitination by inhibiting the binding of the deubiquitinase USP38 to HMGCL. (A) Co‐immunoprecipitation (Co‐IP) and mass spectrometry analyses were performed to identify proteins interacting with both ILF3 and HMGCL. Gene enrichment analysis was performed to identify proteins belonging to the “ubiquitin‐protein ligase” category. (B) Score Sequest HT and gene enrichment of the candidates. (C) Co‐IP assay performed to show the interaction between HMGCL and USP38 in Huh7 and HepG2 cells. (D) Huh7 and HepG2 cells were transfected with USP38 wild‐type or USP38‐CAHA (inactive mutant) plasmids, and Co‐IP and Western blotting were performed to measure endogenous HMGCL ubiquitination after treatment with 5 *μ*M MG132. (E) Huh7 and HepG2 cells were transfected with siRNA or ILF3‐overexpressing plasmids. Western blot assay was performed to measure the effect of ILF3 on USP38 and HMGCL. Additional Co‐IP assay was performed to measure the protein binding capacity between USP38 and HMGCL.

To further identify the potential mediators between ILF3 and HMGCL, we performed immunoprecipitation and mass spectrometry to identify proteins that bind to HMGCL. Bioinformatic analysis of these proteins revealed 112 proteins that could interact with both ILF3 and HMGCL. TRIM36, USP38, and SYVN1 were classified into the ubiquitin‐protein ligase category by gene enrichment analysis (Figure 6). A USP38 had the highest matching score in mass spectrometry (Figure 6). B Co‐IP assays indicated that USP38 interacts with HMGCL in Huh7 and HepG2 cells (Figure 6). C To determine whether USP38 regulates the ubiquitination of endogenous HMGCL, we constructed a catalytically inactive mutant plasmid for USP38 (USP38‐CAHA mutant) [15]. We transfected USP38 and USP38‐CAHA mutant plasmids into HepG2 cells. Ubiquitination assays showed that the ubiquitination level of HMGCL was significantly decreased in cells transfected with USP38 but not in those transfected with the USP38‐CAHA mutant (Figure 6). D Thus, we speculated that USP38 is a deubiquitinating enzyme for HMGCL. Furthermore, we hypothesized that ILF3 competitively binds to HMGCL via USP38, promoting HMGCL ubiquitination and degradation. To test this hypothesis, we transfected HepG2 cells with various concentrations of siRNAs targeting ILF3. WB showed that HMGCL expression gradually increased with increasing concentrations of ILF3‐targeting siRNAs. However, the expression levels of USP38 remained unchanged. Immunoprecipitation assays uncovered that the binding capacity of USP38 to HMGCL rose progressively with reduced ILF3 expression. Furthermore, ILF3 overexpression abrogated the interaction between HMGCL and USP38 (Figure 6). E Collectively, these findings indicate that ILF3 destabilizes HMGCL by competitively binding to USP38, thereby fostering its ubiquitination and degradation.

## 4. Discussion

ILF3 is recognized as an RNA‐stabilizing protein across multiple tumor types [23, 24]. Previous studies have demonstrated that ILF3 is involved in cell cycle regulation, angiogenesis, and RNA stabilization. To further investigate the molecular mechanism by which ILF3 promotes proliferation and metastasis in HCC, we conducted this study. Our study noted significant ILF3 upregulation in both HCC tissues and cell lines, further revealing that ILF3 overexpression correlated with unfavorable prognosis in HCC patients. In addition, we confirmed that ILF3 promotes HCC cell proliferation and metastasis in this study. This demonstrates that ILF3 plays a critical role in regulating HCC progression. In this study, we found a novel mechanism in which ILF3 could interact with HMGCL. Further molecular mechanisms underlying ILF3‐driven HCC proliferation and migration involve the modulation of HMGCL degradation. Our data verified that USP38 is a key regulator that interacts with HMGCL and mediates its deubiquitination. Importantly, ILF3 disrupted the USP38–HMGCL interaction and reduced the stability of HMGCL. Collectively, ILF3 regulates cell proliferation and metastasis by disrupting the interaction between HMGCL and USP38 in HCC.

There have been some studies showing that HMGCL is involved in proliferation and metabolic regulation in cancer. Previous studies have shown that HMGCL activates the MEK‐ERK pathway through an acetoacetic acid–dependent mechanism by inducing OCT1 in melanoma [25]. HMGCL was also found to be overexpressed in the tumor stroma of breast cancer [26]. Another study revealed that it accelerates the production of reactive oxygen species (ROS) and suppresses cell growth and metastasis in nasopharyngeal carcinoma. Of note, HMGCL modulates H3K9 acetylation via *β*‐OHB and triggers ferroptosis in HCC cells [27–29], highlighting its role as a novel, critical suppressor of HCC progression. Our study identified that ILF3 suppresses HMGCL by disrupting the interaction between HMGCL and its deubiquitinase USP38. USP38 was shown to participate in regulating the stability of HMGCL in this study. A previous study also found that USP38 acts as a deubiquitinase implicated in tumorigenesis (including tumor stemness, chemoresistance, and cell proliferation/migration) [30–33]. Prior work has demonstrated that USP38 overexpression impairs glioma cell viability and migration. It was reported that USP38 also regulates migration, invasion, and metastasis in bladder cancer. Here, immunoprecipitation combined with mass spectrometry confirmed USP38 as an interacting partner of both ILF3 and HMGCL. Further ubiquitination assays validated USP38’s role in HMGCL deubiquitination, indicating that the USP38–HMGCL axis is essential for HCC progression.

**Figure 7:**
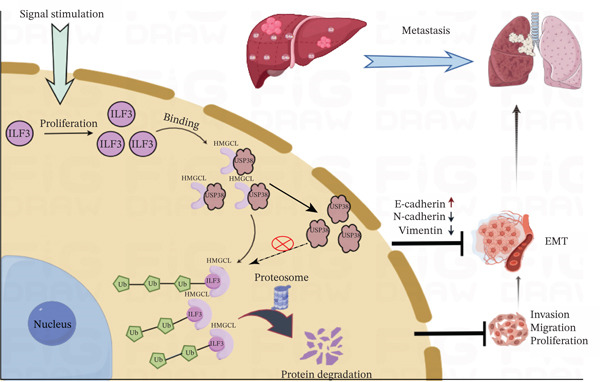
Working model of ILF3 facilitating the proliferation and metastasis of HCC. Deubiquitinase USP38 interacts with HMGCL and contributes to HMGCL deubiquitination. ILF3 is elevated in HCC and facilitates the interaction with HMGCL. ILF3 competitively antagonizes the interaction between USP38 and HMGCL, thereby reducing USP38‐mediated HMGCL deubiquitination and decreasing HMGCL protein abundance. Finally, ILF3 promotes cell proliferation and metastasis in HCC.

Taken together, our study elucidates that ILF3 is upregulated in HCC and is significantly associated with HCC prognosis. Moreover, we found that ILF3 promotes cell proliferation and metastasis in vivo and in vitro. Mechanistically, ILF3 disrupts the USP38–HMGCL interaction, promoting HMGCL ubiquitination and degradation to drive HCC progression (Figure 7). These findings suggest that targeting the ILF3–USP38–HMGCL axis may represent a promising therapeutic strategy for HCC.

## 5. Conclusion

Our study demonstrates that ILF3 is significantly upregulated in HCC tissues and is associated with poor prognosis. We show that ILF3 knockdown suppresses HCC cell proliferation, migration, and metastasis. Mechanistically, we reveal that ILF3 binds to HMGCL, enhances its ubiquitination, and promotes its degradation by competitively antagonizing the interaction between HMGCL and the deubiquitinase USP38. Importantly, overexpression of HMGCL partially reverses the oncogenic effects of ILF3, confirming the functional relevance of this pathway.

## Author Contributions

Qingqing Luo and Lei Xiao contributed equally to this work.

## Funding

This study was supported by the National Natural Science Foundation of China (10.13039/501100001809, Grant No. 82460489) and the Natural Science Foundation of Hunan Province (10.13039/501100004735, Grant Nos. 2025JJ60776 and W202443286).

## Ethics Statement

This study involved human participants and was approved by the Ethics Committee of the First Affiliated Hospital of Guangxi Medical University. All patients provided written informed consent. Animal experiments were reviewed and approved by the Animal Care and Use Committee of Guangxi Medical University.

## Conflicts of Interest

The authors declare no conflicts of interest.

## Supporting Information

Additional supporting information can be found online in the Supporting Information section.

## Supporting information


**Supporting Information 1** Figure S1.

**Supporting Information 2** Table S1.

**Supporting Information 3** Table S2.

## Data Availability

The data that support the findings of this study are available on request from the corresponding authors. The data are not publicly available due to privacy or ethical restrictions.
