# Supplementary material for: ILF3 Regulates Cell Proliferation and Metastasis by Competitively Antagonizing the Interaction Between HMGCL and USP38 in Hepatocellular Carcinoma
Source: Hum Mutat. 2026 Apr 29;2026:2654435. doi: 10.1155/humu/2654435 (PMC13125944; doi:10.1155/humu/2654435)
Supplement: Supplementary file 1 — Supporting Information 1 Figure S1. [file HUMU-2026-2654435-s001.docx]

**
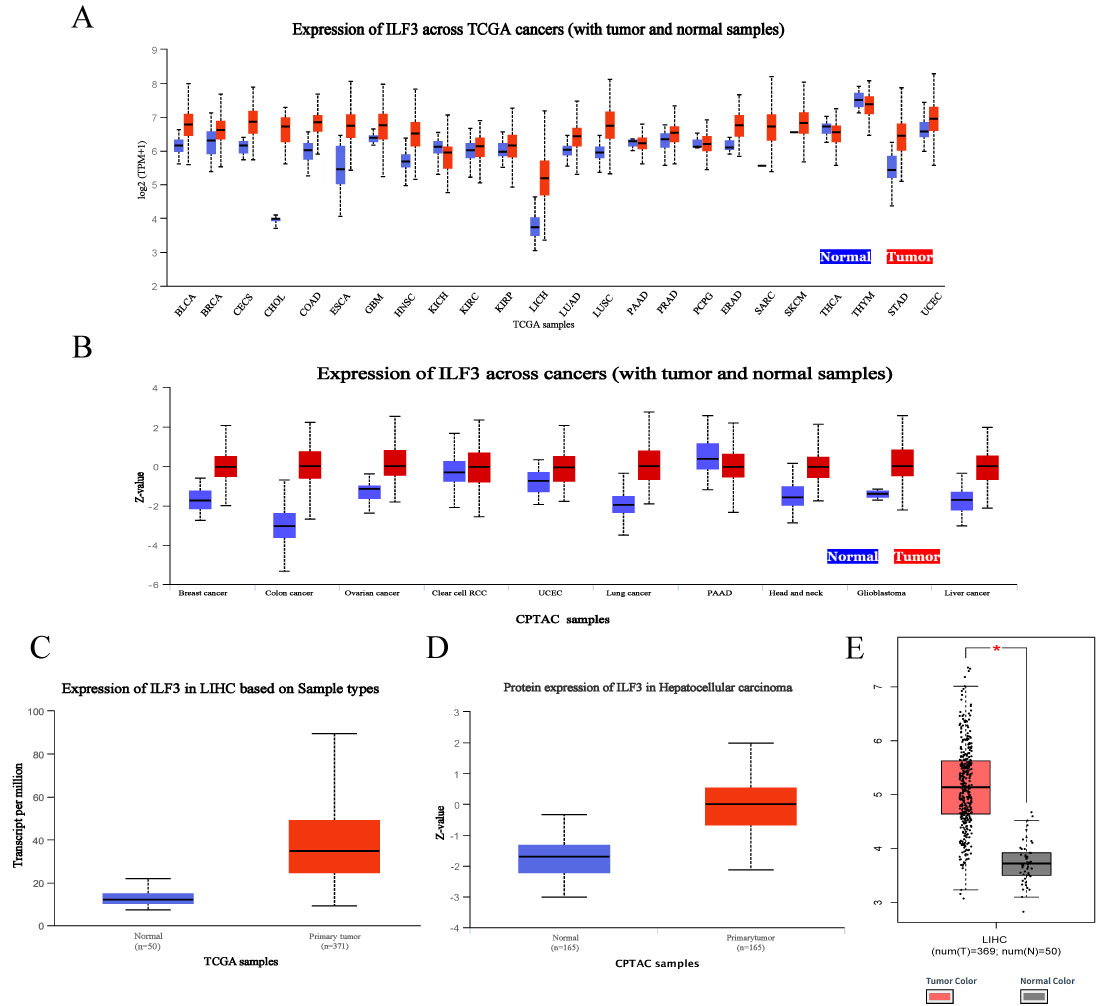
**

**Supplementary Figure 1: ILF3 was significant up-regulatedand in HCC.**

**A**: The expression of ILF3 was up-regulatedand in Pan-cancer according to UALCAN database based on TCGA dataset; **B**: The protein level of ILF3 was up-regulatedand in Pan-cancer according to UALCAN database based on CPATC dataset; **C**:The expression of ILF3 was up-regulatedand in HCC based on TCGA dataset; **D**: The protein expression level of ILF3 was up-regulatedand in HCC based on CPTAC dataset;E: The expression of ILF3 was up-regulatedand in GEPIA dataset.
