# Supplementary material for: ILF3 Regulates Cell Proliferation and Metastasis by Competitively Antagonizing the Interaction Between HMGCL and USP38 in Hepatocellular Carcinoma
Source: Hum Mutat. 2026 Apr 29;2026:2654435. doi: 10.1155/humu/2654435 (PMC13125944; doi:10.1155/humu/2654435)
Supplement: Supplementary file 2 — Supporting Information 2 Table S1. [file HUMU-2026-2654435-s002.docx]

**Supplementary Table 1. Antibodies used in the study**

| **Name** | **Company** | **Catalog Number** | **Antibody solubility** |
| --- | --- | --- | --- |
| ILF3 (Rabbit) | Proteintech | 19887-1-AP | 1:1000 |
| GAPDH (Mouse) | Proteintech | 60004-1-Ig | 1:5000 |
| N-caderin (Rabbit) | CST | #13116 | 1:1000 |
| E-caderin (Rabbit) | CST | #3195 | 1:1000 |
| Vimentin (Rabbit) | Abcam | Ab92547 | 1:5000 |
| HMGCL (Rabbit) | Abcam | Ab197022 | 1:1000 |
| Normal Rabbit IgG (Mouse) | CST | #2729 | 1-5ug for IP |
| Ubiquitin (Mouse) | CST | #3936 | 1:1000 |
| USP38 (Rabbit) | Abcam | Ab72244 | 1:4000 |
