# Supplementary material for: ILF3 Regulates Cell Proliferation and Metastasis by Competitively Antagonizing the Interaction Between HMGCL and USP38 in Hepatocellular Carcinoma
Source: Hum Mutat. 2026 Apr 29;2026:2654435. doi: 10.1155/humu/2654435 (PMC13125944; doi:10.1155/humu/2654435)
Supplement: Supplementary file 3 — Supporting Information 3 Table S2. [file HUMU-2026-2654435-s003.docx]

**Supplementary Table 2. Primers for RT-qPCR**

| **Name** | **Primer Sequence** |
| --- | --- |
| ILF3-F | ATGGCTGGCGGCGTGTTT |
| ILF3-R | TCAGCTTCCTCGGCGTTC |
| HMGCL-F | CAGGTGCTGGAGAAGGACCT |
| HMGCL-R | TCCAGGTCCACGTCCTTGTA |
| GAPDH-F | GGAAGCTTGCATCAATGGAAATC |
| GAPDH-R | TGATGACCCTTTTGGCTCCC |
